# Metabolomic Profiling of the White, Violet, and Red Flowers of *Rhododendron schlippenbachii* Maxim.

**DOI:** 10.3390/molecules23040827

**Published:** 2018-04-04

**Authors:** Chang Ha Park, Hyeon Ji Yeo, Nam Su Kim, Ye Eun Park, Soo-Yun Park, Jae Kwang Kim, Sang Un Park

**Affiliations:** 1 Department of Crop Science, Chungnam National University, 99 Daehak-Ro, Yuseong-gu, Daejeon 34134, Korea; parkch804@gmail.com (C.H.P.); guswl7627@gmail.com (H.J.Y.); kns917555@naver.com (N.S.K.); yeney1996@cnu.ac.kr (Y.E.P.); 2 National Institute of Agricultural Sciences, Rural Development Administration, Wanju-gun, Jeonbuk 54875, Korea; psy22@korea.kr; 3 Division of Life Sciences and Bio-Resource and Environmental Center, Incheon National University, Incheon 406-772, Korea

**Keywords:** *Rhododendron schlippenbachii* Maxim., anthocyanin, metabolomic profiling

## Abstract

*Rhododendron schlippenbachii* Maxim. is a garden plant that is also used for natural medicines as a consequence of the biological activities of its diverse metabolites. We accordingly profiled two anthocyanins and 40 primary and secondary metabolites in the three different colored flowers. The major anthocyanins found in the flowers were cyanidins. The red flowers exhibited the highest accumulation of anthocyanins (1.02 ± 0.02 mg/g dry weight). Principal component analysis was applied to the GC‒TOFMS data. The levels of key tricarboxylic acid cycle intermediates in red flowers, such as succinic acid, fumaric acid, and malic acid, were found to be highly significantly different (*p* < 0.0001) from those in the flowers of other colors. In this study, we aimed to determine metabolite interactions and phenotypic variation among white, violet, and red flowers of *R. schlippenbachii* by using gas chromatography time-of-flight mass spectrometry (GC‒TOFMS) and high-performance liquid chromatography (HPLC).

## 1. Introduction

**Figure 1:**
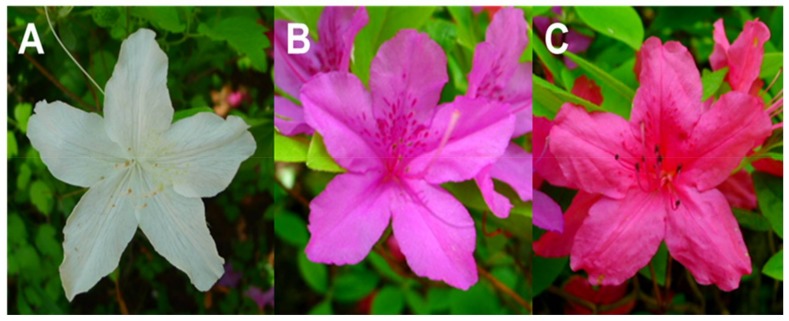
Three different colored flowers of *Rhododendron schlippenbachii*. (**A**) white, (**B**) violet, (**C**) red.

*Rhododendron*, one of the largest genera of shrubs, is distributed throughout most of the Northern hemisphere and the various species are used extensively as garden plants because of their variety of flower colors and evergreen leaves [1,2]. Moreover, numerous species in this genus have been used in traditional medicine in Europe, China, and North America, even though rhododendrons are a source of contaminated honey causing intoxication [2,3]. Such applications are based on the large number of phytochemicals that have diverse biological properties, including antimicrobial [4], anti-inflammatory [5], antidiabetic [6] and antioxidative activities [7]. In addition to the attractive flower colors that make it a popular garden plant (Figure 1), *Rhododendron schlippenbachii* Maxim. also has the potential to be a good source of natural medicines, as previous studies have reported antihyperglycemic, cholinesterase inhibitor, and antioxidant activities [8,9,10]. 

Flower colors contribute to attracting insects and animals for pollination or seed dispersal and to protect plants from the damage caused by UV and visible light [11]. Anthocyanins, a group of flavonoids, are non-toxic water-soluble pigments responsible for a range of orange, pink, red, violet and blue colors in flowers. Among the large number of anthocyanins present in nature, six common anthocyanidins (cyanidin, malvidin, delphinidin, pelargonidin, petunidin, and peonidin) are widespread in vascular plants [11,12,13]. Dey and Harborne (1989) reported that cyanidin, pelargonidin, and delphinidin derivatives, such as non-methylated anthocyanidins, are the most common [14]. In vegetables and fruits, cyanidins, which are involved in color determination (i.e., reddish colors) occur more commonly than the other anthocyanidins [15,16]. Cyanidins have been shown to have a variety of biological properties, including prevention of inflammation and damage caused by UV in liposomes [17,18], suppression of oxidative damage in human red blood cells [19], and anticarcinogenic [20], antidiabetic [21], antioxidant [18], and antimutagenic effects [22].

Metabolomic profiling provides comprehensive information on a diversity of cell- or organism-specific responses to different biological or environmental conditions, including the identification and quantitation of low molecular weight metabolites found in biological systems [23,24,25]. Gas chromatography–mass spectrometry is regarded as a useful analytical tool for metabolic profiling, as it facilitates a broad coverage of chemical compound classes and stable retention time and the capacity to detect a relatively broad range of chemical compounds through robust optimized protocols for sample preparation and equipment operation [25,26,27]. In particular, gas chromatography time-of-flight mass spectrometry (GC‒TOFMS) enables enhanced deconvolution or reduced execution time for complex mixtures, resulting from fast scan times (10–50 scans per second) and greater mass accuracy [27,28].

To date, however, there has been no comprehensive description of the primary and secondary metabolites in *R. schlippenbachii*. Thus, in this study, we used a combination of high-performance liquid chromatography (HPLC), GC‒TOFMS, and chemometrics to analyze the variation in color phenotype among three different colored flowers of *R. schlippenbachii,* with the aim of gaining a comprehensive understanding of the anthocyanins and hydrophilic metabolites of this plant.

## 2. Results and Discussion

### 2.1. Anthocyanin Analysis

The HPLC analysis revealed a total of two anthocyanins, cyanidin-3,5-diglucoside and cyanidin-3-sambubioside. The red flowers exhibited the highest amount of total anthocyanin (1.02 ± 0.02 mg/g dry weight (wt.)), followed by violet flowers (0.30 ± 0.01 mg/g dry wt.). In contrast, no anthocyanins were detected in the white flowers. Additionally, comparison of individual cyanidins showed that red flowers contained the highest levels of each of the cyanidins detected (Table 1). 

**Figure 2:**
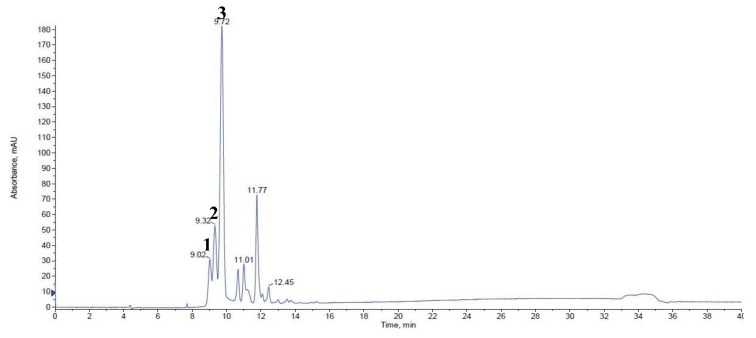
LC-MS TIC spectrum of anthocyanins in red flowers of *Rhododendron schlippenbachii*. Peak Identification: 1, cyanidin-3,5-diglucoside; 2, cyanidin-3,5-diglucoside; 3, cyanidin-3-sambubioside, Refer to Table 1 for the identification of each numbered peak.

In Figure S1, Figure 2, and Table 1, cyanidin-3,5-diglucoside (Peak 1), cyanidin-3,5-diglucoside (peak 2), and cyanidin-3-sambubioside (peak 3) were identified by LC–MS/MS analysis, which detected positively monocharged molecular ions at *m*/*z* 611, 611, and 581, respectively. The different retention times of cyanidin-3,5-diglucoside may be attributed to anomeric forms of glucose. The major fragments for their peaks appeared at *m*/*z* 287 (corresponding to cyanidin) and 499 (cyanidin-3-glucoside). It is accordingly suggested that cyanidins are the major anthocyanins ubiquitous in the flowers of *R. schlippenbachii*.

### 2.2. Metabolic Profiles Using GC-TOFMS Analysis

**Figure 3:**
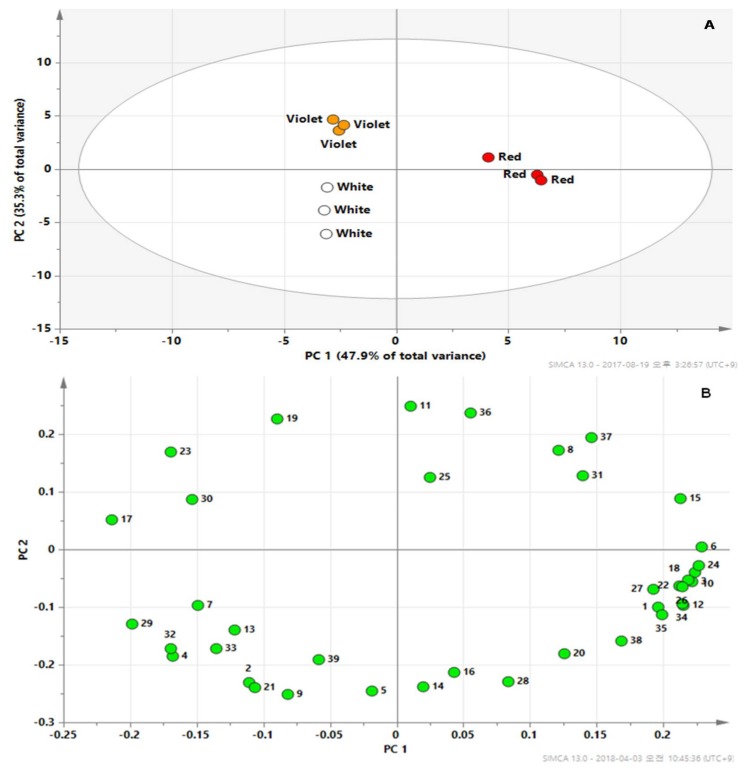
Scores (**A**) and loading (**B**) plots of principal components 1 and 2 of the principal component analysis results for polar metabolite data obtained for the red, violet, and white flowers of *R. schlippenbachii*. 1, lactic acid; 2, valine; 3, glycolic acid; 4, serine; 5, ethanolamine; 6, glycerol; 7, proline; 8, nicotinic acid; 9, glycine; 10, succinic acid; 11, glyceric acid; 12, fumaric acid; 13, threonine; 14, β-alanine; 15, malic acid; 16, aspartic acid; 17, pyroglutamic acid; 18, 4-aminobutyric acid; 19, threonic acid; 20, glutamic acid; 21, phenylalanine; 22, p-hydroxybenzoic acid; 23, xylose; 24, asparagine; 25, vanillic acid; 26, glutamine; 27, citric acid; 28, quinic acid; 29, fructose; 30, glucose; 31, mannose; 32, inositol; 33, ferulic acid; 34, tryptophan; 35, sinapinic acid; 36, sucrose; 37, maltose; 38, trehalose; 39, raffinose.

To the best of our knowledge, no previous studies have performed a comprehensive analysis of primary and secondary metabolites in the flowers of *R. schlippenbachii* through GC–TOFMS-based metabolic profiling, although the amino acid contents in *R. schlippenbachii* flowers have been reported [29]. Therefore, in the present study, we identified and quantified the low molecular weight hydrophilic compounds in three different colored flowers of *R. schlippenbachii* using GC–TOFMS. Peak determination and data processing were performed as described in the Materials and Methods. In total, 39 hydrophilic compounds were detected in the flower samples. The quantitation data of 39 metabolites were normalized to the signal intensity of the internal standard and then subjected to PCA to explore the data structure (Figure 3).

The results of PCA of metabolic profiles clearly showed the lack of marked variance among individual cultivars and distinguished three cultivars. Two principal components of the score plot explained 83.2% of the total variance (component 1, 47.9%; component 2, 35.3%). Component 1 resolved the separation of red flowers from the other flower samples. Additionally, the metabolomes of violet flowers and white flowers were separated by component 2 above and below. In comparison with the metabolic loadings in component 1 and 2, the significant compounds of component 1 in the loading plot were glycerol, asparagine, succinic acid, glycolic acid, GABA, fumaric acid, tryptophan, malic acid, glutamine, and *p*-hydroxybenzoic acid, for which the eigenvector values were greater than 0.20. The loading indicated that the levels of the main intermediates of the tricarboxylic acid (TCA) cycle, including succinic acid, fumaric acid, and malic acid, were higher in red flowers compared with those measured in white and violet flowers (Figure S2). Red flower also had higher levels of asparagine than white and violet flowers. Park (2016) [29] reported that red flowers exhibited 3.70- and 2.48-fold higher levels of asparagine than those of the violet and white flowers of *R. schlippenbachii*. Similarly, it has previously been shown that the peel of red ‘Anjou’ has higher activities of asparagine synthetase than green ‘Anjou’ peel [30].

**Figure 4:**
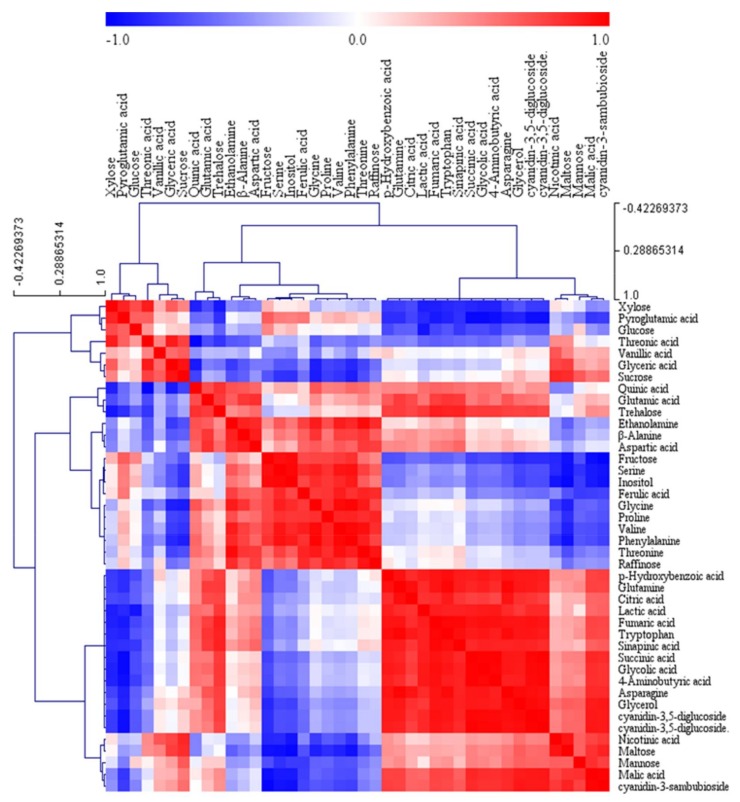
Correlation matrix of 42 metabolites from three different colored flowers of *R*. *schlippenbachii*. Each square indicates the Pearson correlation coefficient of a pair of compounds. The value of correlation coefficient is represented based on the intensity of the red and blue colors. Secondary metabolites were marked by a yellow box.

Furthermore, Pearson correlation analysis was used to analyze the correlation among 42 metabolites, including anthocyanins, in the different colored flowers. A hierarchical clustering analysis of the Pearson correlation coefficients was performed to visualize the comprehensive relationship between these metabolites (Figure 4). Among TCA organic acids, citric acid was positively correlated with succinic acid (r = 0.75159, *p* = 0.0195) and fumaric acid (r = 0.82593, *p* = 0.0061). This finding was consistent with the results from PCA. Most Pearson correlation coefficients between TCA intermediates and phenolic compounds, including vanillic acid, *p*-hydroxybenzoic acid, quinic acid, sinapinic acid, cyanidin-3,5-diglucoside, and cyanidin-3-sambubioside, were higher than 0.7. Likewise, strong positive relationships were detected between anthocyanins and sugars including glycerol, galactose, mannitol, mannose, maltose, and trehalose. 

Carbohydrates are the most abundant metabolites in the white, violet, and red flowers. A comparison of the sugar levels between the different colored flowers indicated that the value of total carbohydrates in the red flowers was lower than that in the other flowers (Figure S3). In contrast, the total levels of amino acids were similar to each other. In detail, the larger pool of alanine, which is amino donors in photorespiration, reflected that the higher levels of serine (8.99-fold) and glycine (2.07-fold), which are photorespiratory intermediates, in white flowers. Furthermore, the oxaloacetic acid family of amino acids, including aspartic acid, asparagine, and threonine, were generally higher in red flowers than in white and violet flowers. Among the four intermediates of the TCA cycle that were identified and quantitated, the red flowers contained the largest pools of malic acid, fumaric acid, and succinate, whereas the pool of citric acid was comparable with that of the white and violet flowers, which is consistent with the higher quantity of aspartic acid.

Cyanidin, one of the major anthocyanins, is responsible for red-purple color and is a dominant anthocyanin aglycone present in plants [31]. In this study, anthocyanin analysis for the different colored flowers of *R. schlippenbachii* confirmed that the color variation between the flowers was affected by anthocyanin pigments, including cyanidin-3,5-diglucoside and cyanidin-3-sambubioside, and that a higher accumulation of anthocyanins confers deeper reddish colors. This is consistent with the findings of a previous study that examined the role of anthocyanin in the plant pigmentation [32]. Furthermore, previous studies have reported that cyanidin-3-sambubioside was identified as the pink pigment [33,34] and detected in red raspberries and elderberry juice [35,36]. Similarly, ribberry [37] and red cabbage contain cyanidin-3,5-diglucoside as the predominant anthocyanin [38]. 

Primary metabolism is known to comprise reactions essential for plant survival, involving the production and use of a range of molecules, including nucleic acids, amino acids, carbohydrates, fatty acids, and biopolymers. In contrast, secondary metabolism is defined as biochemical reactions playing a main role in plant defense. Moreover, primary metabolites, substances generated from primary metabolism, are concerned with pathways related to a diversity of secondary metabolites [38,39,40,41,42].

Carbohydrates function as metabolic precursors and energy sources in plants. The total production of carbohydrates, represented by glycerol, xylose, trehalose, fructose, galactose, glucose, mannose, mannitol, inositol, sucrose, raffinose, and maltose in the red flowers of *R. schlippenbachii* was lower than that in the white and violet flowers at *p* < 0.05, reflecting the demand for energy and carbon to promote anthocyanin glycosylation and production. These findings are consistent with those of a previous study that showed that the total amount of carbohydrates for anthocyanin production in purple kohlrabi was higher than that in green kohlrabi [43]. Zulak et al. (2008) also determined that the promotion of alkaloid biosynthesis in cell cultures of *Papaver somniferum* L. supplemented with a fungal elicitor resulted in a more rapid depletion of carbohydrate pools [44]. Furthermore, the higher level of tryptophan in red flowers suggested a greater production of anthocyanins. Previously, brief exposure to UV has been shown to positively affect tryptophan and phenylalanine/tyrosine synthesis, thereby leading to an enhanced accumulation of anthocyanin [45]. 

Flavonol biosynthesis and cellular catabolism are related to intermediates of the TCA cycle [46]. 2-Oxoglutarate is a key molecule of the TCA cycle [47,48] and a mandatory substrate for 2-OG-dependent dioxygenases. Four types of 2-OG-dependent dioxygenases, such as flavonol synthase, flavanone 3-hydroxylase, flavone synthase I, and anthocyanin synthase, have been characterized as main enzymes involved in late stages of flavonoid aglycone formation in flavonoid biosynthesis. According to the Pearson correlation analysis, TCA intermediates were positively correlated with anthocyanins and phenolic compounds. Additionally, PCA analyses showed that red flowers were apparently separated from white and violet flowers samples. Therefore, the higher amount of intermediates of TCA cycle in the red flowers of *R. schlippenbachii* suggested a greater accumulation of anthocyanin, which is consistent with previous findings [49]. 

## 3. Materials and Methods

### 3.1. Plant Materials

White, violet, and red flowers of *R. schlippenbachii* were obtained from an experimental field of Chungnam National University in May 2017 and immediately submerged in liquid nitrogen at −196 °C. The flower samples were subsequently freeze-dried once at −80 °C for 3 days and then ground into a very fine powder.

### 3.2. Anthocyanin Extraction and HPLC Analysis

Anthocyanin HPLC analysis was performed according to the procedure described by Park et al. [50]. Prior to sample injection, 2 mL water/methanoic acid (95:5, *v*/*v*) was added to tubes containing 100 mg of the powdered preparations of the different colored flowers and vortexed for 5 min. Each tube was mildly sonicated for 20 min and then centrifuged at 12,000 rpm and 4 °C for 15 min. The resulting supernatants were passed into 1.8-mL HPLC glass vials through a PTFE hydrophilic syringe filter (diameter 13 mm, pore size 0.45 µm). After injection of 10 μL of each sample into a Perkin–Elmer Flexar HPLC system (Shelton, CT, USA) equipped with a PDA LC detector, anthocyanin separation was carried out on a Synergy 4 μ Polar-RP 80A (250 × 4.6 mm, i.d.) column connected to a Security Guard Cartridges Kit (AQ C18, 4 × 3 mm, i.d.; Phenomenex, Torrance, CA, USA) thermostatically controlled at 40 °C, using mobile phase solvents consisting of solvent (A) water/methanoic acid (95:5, *v*/*v*) and solvent (B) acetonitrile/methanoic acid (95:5, *v*/*v*) at a flow rate of 1 mL/min and detection wavelength of 520 nm. The gradient program was set as follows: 0.0 min, 5% solvent B; 30.00 min, 40% solvent B; 30.10 min, 5% solvent B; 40.00 min, 5% solvent B (total 30 min). The entire procedure was repeated in triplicate. 

### 3.3. LC-MS/MS Analysis for the Quantification of Anthocyanin Contents

For the quantification of anthocyanin contents, we used a liquid chromatography–tandem mass spectrometry (LC‒MS/MS) analysis system consisting of an Agilent 1200 series system coupled to an electrospray ionization mass spectrometer and a 4000 Qtrap LC‒MS/MS system (Applied Biosystems Instrument, Foster City, CA, USA). The LC‒MS/MS operating conditions were as follows: scan range, 100‒1300 *m*/*z*; scan time, 4.80 s; curtain gas, 20.00 psi (N_2_), scan mode, positive ion mode; heating gas temperature, 550 °C; heating gas, 50.00 psi; nebulizing gas, 50.00 psi; ion spray voltage, 5500 V; entrance potential, 10 V; and declustering potential, 100 V. As cyanidin-3,5-diglucoside and cyanidin-3-sambubioside were not commercially available, cyanidin 3-*O*-glucoside (C3G) was used instead. The concentrations of two different anthocyanins were calculated as equivalents of C3G using a standard curve derived from the commercial anthocyanin. Anthocyanin contents were expressed as milligram per gram dry weight (mg/g dry wt.) [51].

### 3.4. GC‒TOFMS Analysis

Hydrophilic metabolites were extracted as described previously [49,52]. Ten milligrams of powdered sample was added to 1 mL of methanol/chloroform/water (2.5:1:1, *v*/*v*/*v*) and adonitol (60 µL, 200 ppm) as an internal standard. Incubation was carried out at 37 °C with a mixing frequency of 1200 rpm for 30 min, using a compact thermomixer (Eppendorf AG, Hamburg, Germany). The mixture was then centrifuged at 16,000× *g* for 3.5 min, and 800 µL of the polar phase was subsequently transferred to a fresh tube, to which 400 µL of distilled water was then added. After centrifugation of the mixed contents at 16,000× *g* for 3.5 min, 900 µL of the supernatant was transferred to a new tube. The water‒methanol phase was dried using a centrifugation concentrator (CC–105; TOMY, Tokyo, Japan) for 2 h, followed by freeze-drying for 16 h. For methoxime derivatization, 80 μL of methoxyamine hydrochloride (20,000 ppm) in pyridine was added and shaken for 90 min at 30 °C. After the addition of 80 μL of *N*-methyl-*N*-trimethylsilyltrifluoroacetamide, the mixtures were incubated for 30 min at 37 °C. GC–TOFMS analysis was performed as described by Park et al. (2017) [43]. The GC-TOFMS analysis conditions were set as follows: split ratio, 1:25; injector temperature, 230 °C; flow rate of helium gas, 1.0 mL/min; mass range, 85–600 *m*/*z*; and detector voltage, 1700 V. The temperature program used was as follows: the initial temperature of 80 °C was held for 2 min, then increased to 320 °C at a rate of 15 °C/min, and held at 320 °C for 10 min. The ion source temperature and transfer line were 200 °C and 250 °C, respectively. Prior to quantitative analysis, ChromaTOF software was utilized for peak detection and automated deconvolution of reference mass spectra. In-house libraries for standard compounds and the NIST database were used to identify the metabolites. Calculations of the concentrations of all analytes were based on the ratio calculated from the peak area of an individual compound/the peak area of the internal standard (adonitol). 

### 3.5. Statistical Analysis

The data from the HPLC and GC–TOFMS were statistically analyzed using Statistical Analysis System software (SAS, system 9.4, 2013; SAS Institute, Inc., Cary, NC, USA). The statistical significance among means was evaluated by Duncan’s Multiple Range Test (DMRT) with a significance level of *p* ≤ 0.05. All data are represented as the mean ± standard deviation of triplicate tests. SIMCA-P (version 13.0; Umetrics, Umeå, Sweden) was used to determine the relationship with regards to similarity between groups of multivariate data acquired from GC–TOFMS [15]. Prior to subjecting the multivariate data to principal component analysis (PCA), data scaling was performed by unit variance scaling. The PCA output was composed of score plots for visualizing the contrast between different colored flowers of *R*. *schlippenbachii* and loading plots to account for the cluster separation. 

## 4. Conclusions

In conclusion, in this study, we determined phenotypic differences between the white, violet, and red flowers of *R*. *schlippenbachii* via comprehensive analysis of the primary and secondary metabolites of the three different colored flowers using GC–TOFMS and HPLC. Anthocyanin pigments are regarded as the main factors determining phenotypic differences in the color of the three different flowers. The red flowers contained high amounts of anthocyanins and low levels of carbohydrates, reflecting the carbon and energy demand. Furthermore, the observed high amounts of TCA intermediates, which are involved in anthocyanin metabolism, were consistent with the high amounts of anthocyanins. The findings of this study accordingly confirm that HPLC- and GC–TOFMS-based metabolite profiling is a suitable approach for determining the metabolic interplay and phenotypic variation among the different colored flowers of *R. schlippenbachii*.

## Figures and Tables

**Table 1:** Anthocyanin contents (mg/g dry wt.) of different colored flowers of *Rhododendron schlippenbachii*.

No. ^1^	Retention Time	Trivial Name	[M + H]^+^ (*m*/*z*)	MS/MS (*m*/*z*)	White	Violet	Red
1	9.02	cyanidin-3,5-diglucoside	611	449/287	ND ^2^	ND	0.11 ± 0.01
2	9.32	cyanidin-3,5-diglucoside	611	449/287	ND	ND	0.21 ± 0.00
3	9.72	cyanidin-3-sambubioside	581	287	ND	0.30 ± 0.01 ^b^	0.70 ± 0.01 ^a^
	Total				ND	0.30 ± 0.01 ^b^	1.02 ± 0.02 ^a^

^1^ No., the elution order of anthocyanins in HPLC analysis. ^2^ ND, not detected. Different letters (^a,b^) differ significantly (*p* < 0.05, ANOVA, DMRT).

## Supplementary Materials

The following are available online. Figure S1: LC-MS spectrum of cyanidin-3,5-diglucoside and cyanidin-3-sambubioside in *Rhododendron schlippenbachii* Maxim. Figure S2: Box plots of metabolites that had eigenvector values greater than 0.2 for component 1 obtained from principal component analysis. The metabolites were significantly different (*p* < 0.0001) between the three different colored flowers of *Rhododendron schlippenbachii* Maxim. Figure S3: Metabolite peak area ratio of different colored flowers of *Rhododendron schlippenbachii*. based on Duncan’s Multiple Range Test (*p* < 0.05) using GC–TOFMS. Figure S4: Selected ion chromatograms of hydrophilic metabolites extracted from red flowers of *R. schlippenbachii* as MO/TMS derivatives separated on a 30 m × 0.25 mm i.d. fused silica capillary column coated with 0.25 μm CP-SIL 8 CB low bleed. The numbers represent the same compounds as for Table S1. Table S1: Metabolites identified in GC-TOFMS chromatograms of red flowers of *Rhododendron schlippenbachii* Maxim.
