# Supplementary material for: Metabolomic Profiling of the White, Violet, and Red Flowers of Rhododendron schlippenbachii Maxim
Source: Molecules. 2018 Apr 4;23(4):827. doi: 10.3390/molecules23040827 (PMC6017568; doi:10.3390/molecules23040827)
Supplement: Supplementary file 1 [file molecules-23-00827-s001.pdf]

Figure S1. LC-MS spectrum of cyanidin-3,5-diglucoside and cyanidin-3-sambubioside in *Rhododendron schlippenbachii* Maxim.

Figure S1a. LC-MS spectrum of cyanidin-3,5-diglucoside in *Rhododendron schlippenbachii* Maxim.

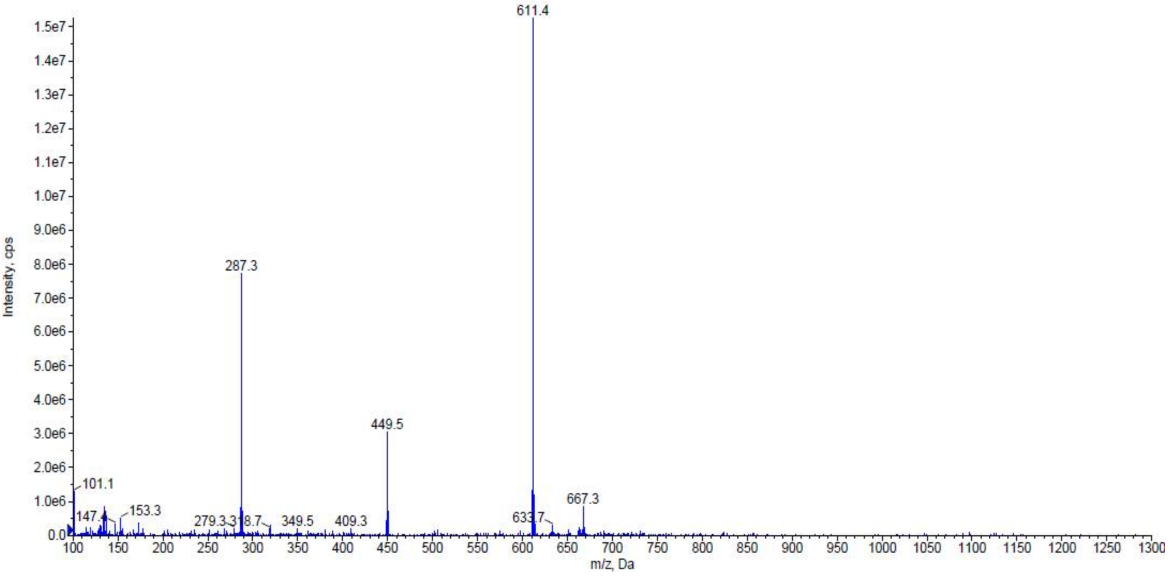

Figure S1b. LC-MS spectrum of cyanidin-3,5-diglucoside in *Rhododendron schlippenbachii* Maxim.

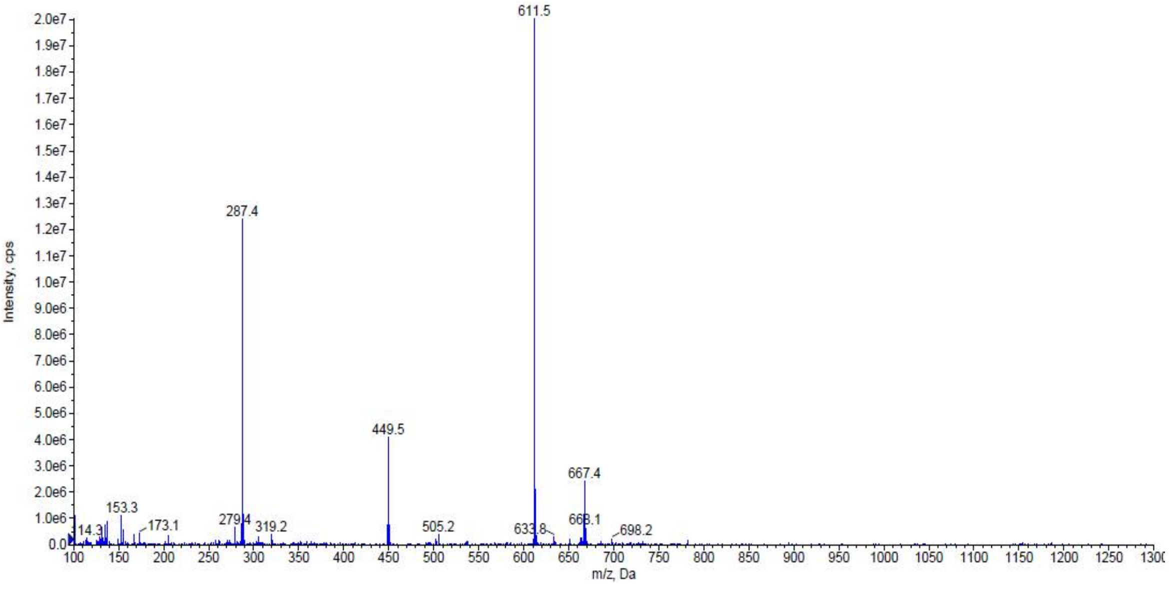

Figure S1c. LC-MS spectrum of cyanidin-3-sambubioside in *Rhododendron schlippenbachii* Maxim.

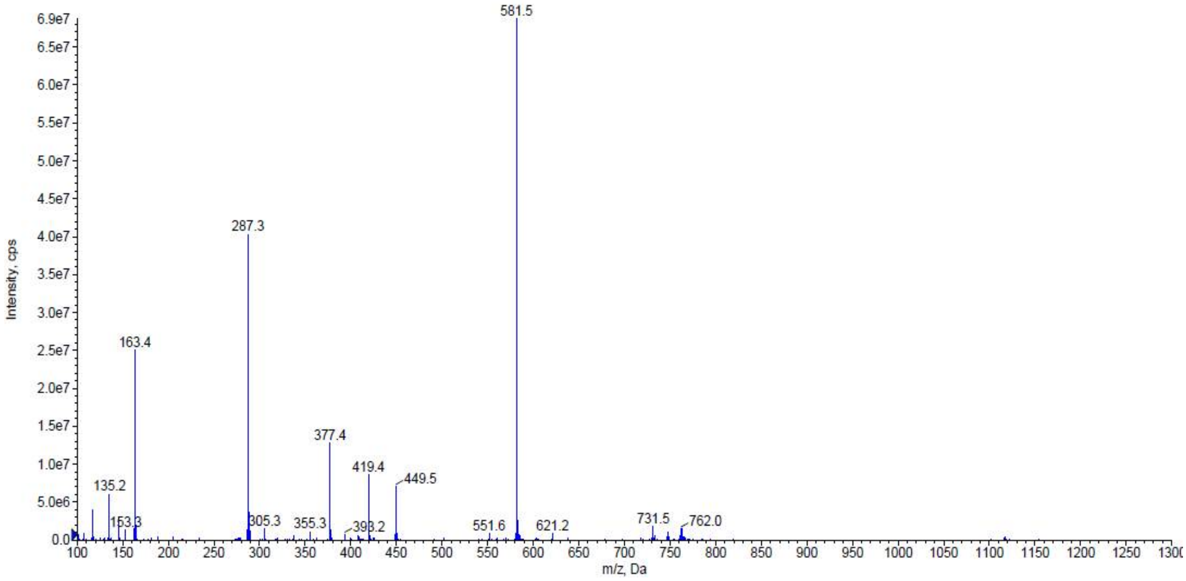

Figure S2. Box plots of metabolites that had eigenvector values greater than 0.2 for component 1 obtained from principal component analysis. The metabolites were significantly different ($p < 0.0001$) between the three different colored flowers of *Rhododendron schlippenbachii* Maxim.

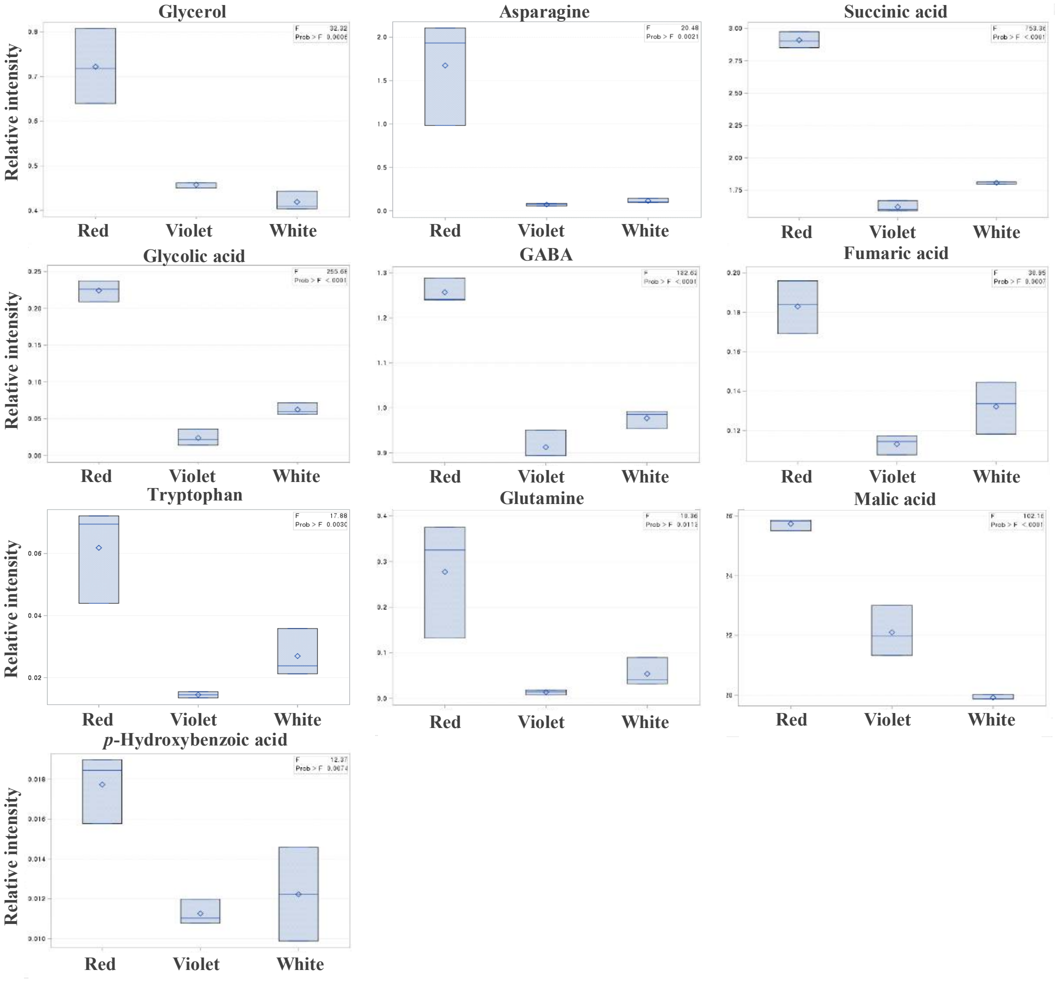

Figure S3. Metabolite peak area ratio of different colored flowers of *Rhododendron schlippenbachii*. Maxim based on Duncan's Multiple Range Test ($p < 0.05$) using GC–TOFMS.

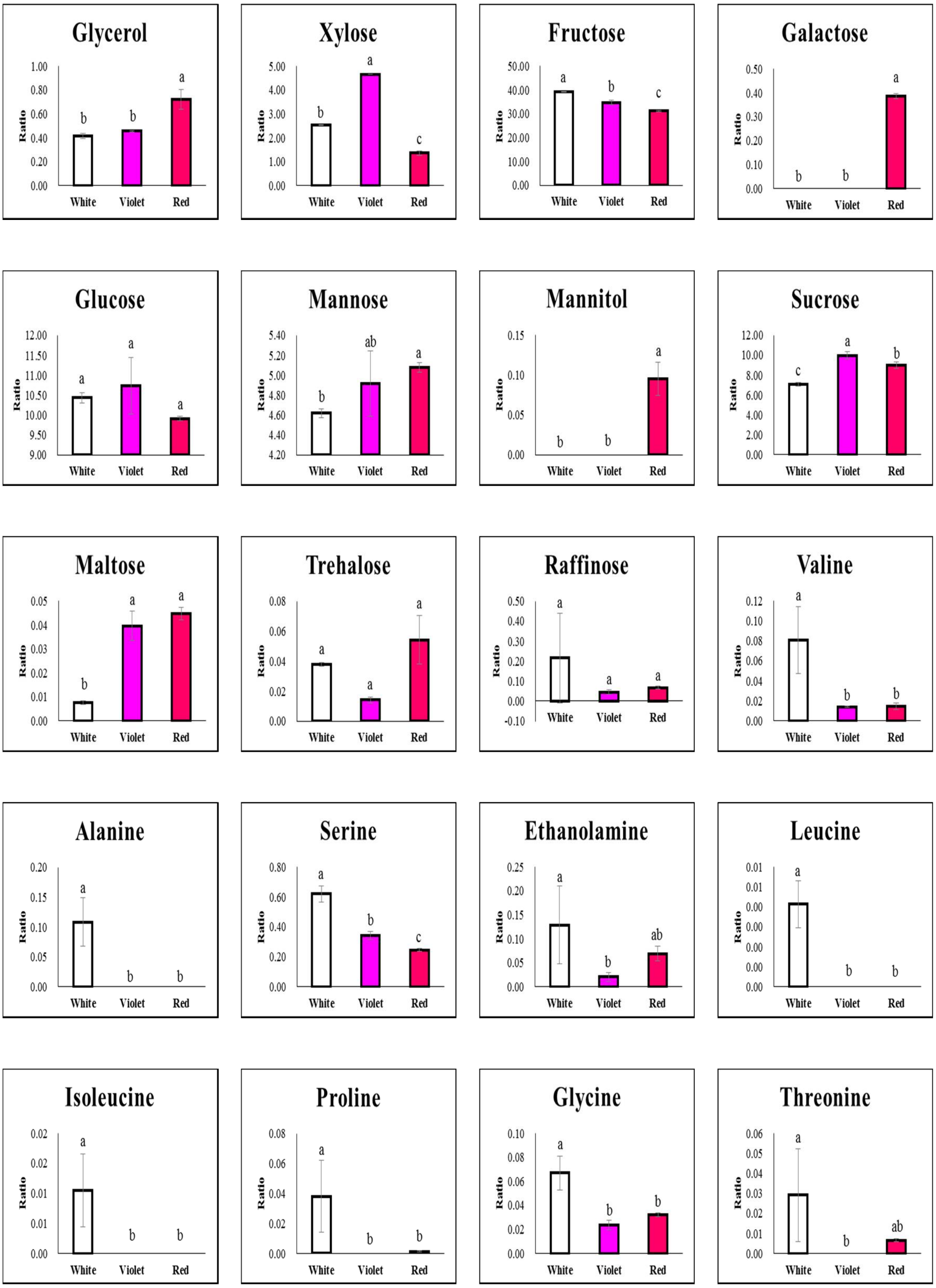

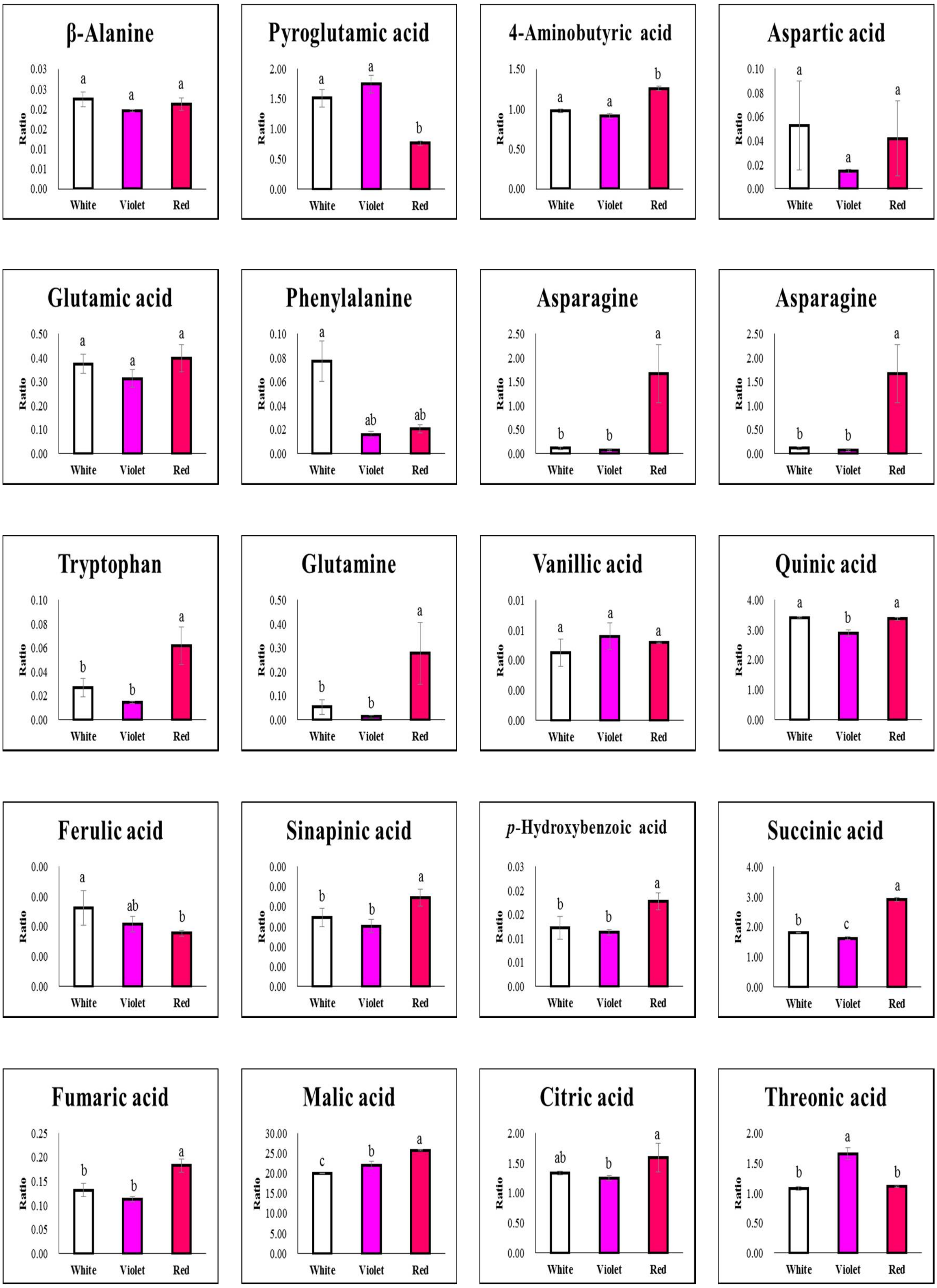
β-Alanine
Ratio
0.00 0.01 0.01 0.02 0.02 0.03 0.03
White Violet Red
a a a
Pyroglutamic acid
Ratio
0.00 0.50 1.00 1.50 2.00
White Violet Red
a a b
4-Aminobutyric acid
Ratio
0.00 0.50 1.00 1.50
White Violet Red
a a b
Aspartic acid
Ratio
0.00 0.02 0.04 0.06 0.08 0.10
White Violet Red
a a a
Glutamic acid
Ratio
0.00 0.10 0.20 0.30 0.40 0.50
White Violet Red
a a a
Phenylalanine
Ratio
0.00 0.02 0.04 0.06 0.08 0.10
White Violet Red
a ab ab
Asparagine
Ratio
0.00 0.50 1.00 1.50 2.00 2.50
White Violet Red
b b a
Asparagine
Ratio
0.00 0.50 1.00 1.50 2.00 2.50
White Violet Red
b b a
Tryptophan
Ratio
0.00 0.02 0.04 0.06 0.08 0.10
White Violet Red
b b a
Glutamine
Ratio
0.00 0.10 0.20 0.30 0.40 0.50
White Violet Red
b b a
Vanillic acid
Ratio
0.00 0.00 0.00 0.01 0.01
White Violet Red
a a a
Quinic acid
Ratio
0.00 1.00 2.00 3.00 4.00
White Violet Red
a b a
Ferulic acid
Ratio
0.00 0.00 0.00 0.00 0.00
White Violet Red
a ab b
Sinapinic acid
Ratio
0.00 0.00 0.00 0.00 0.00 0.00 0.00
White Violet Red
b b a
p-Hydroxybenzoic acid
Ratio
0.00 0.01 0.01 0.02 0.02 0.03
White Violet Red
b b a
Succinic acid
Ratio
0.00 1.00 2.00 3.00 4.00
White Violet Red
b c a
Fumaric acid
Ratio
0.00 0.05 0.10 0.15 0.20 0.25
White Violet Red
b b a
Malic acid
Ratio
0.00 5.00 10.00 15.00 20.00 25.00 30.00
White Violet Red
c b a
Citric acid
Ratio
0.00 0.50 1.00 1.50 2.00
White Violet Red
ab b a
Threonic acid
Ratio
0.00 0.50 1.00 1.50 2.00
White Violet Red
b a b

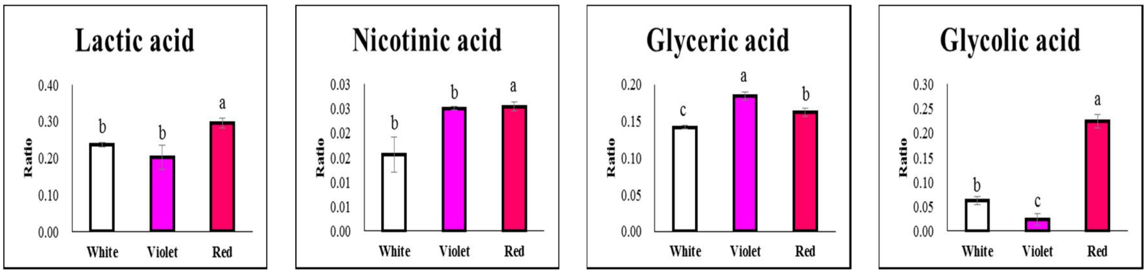

Figure S4. Selected ion chromatograms of hydrophilic metabolites extracted from red flowers of R. schlippenbachii as MO/TMS derivatives separated on a 30 m × 0.25 mm i.d. fused silica capillary column coated with 0.25 μm CP-SIL 8 CB low bleed. The numbers represent the same compounds as for Table S1.

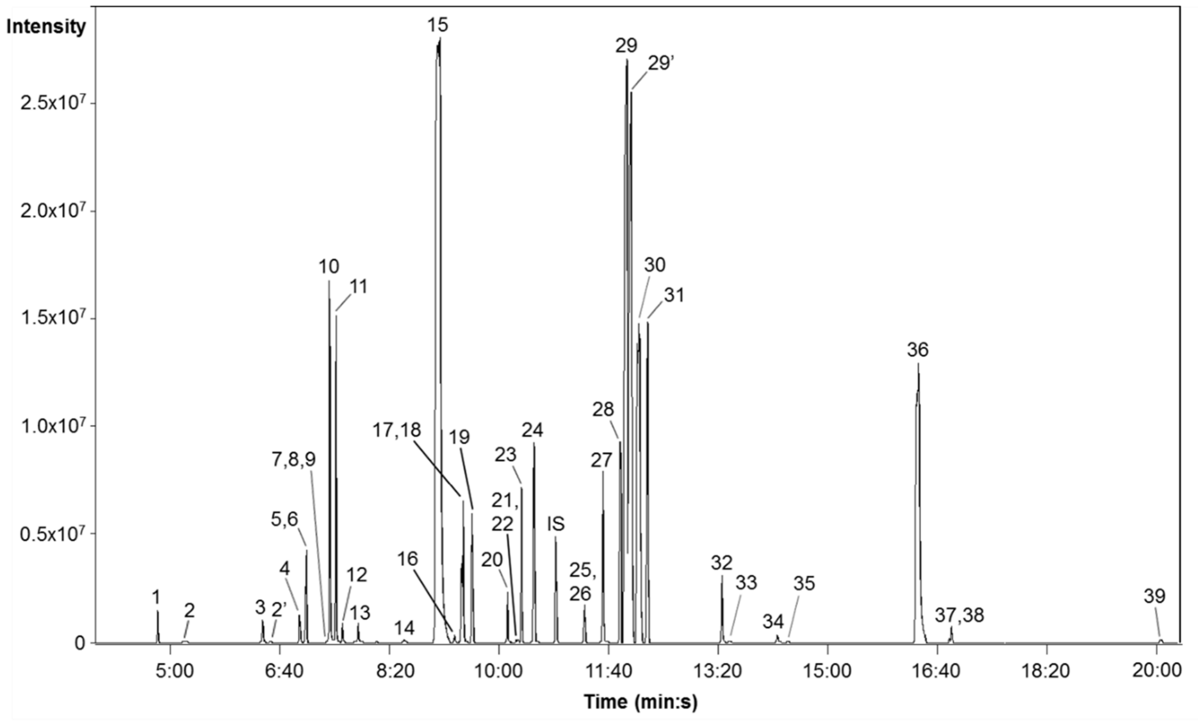

Table S1. Metabolites identified in GC-TOFMS chromatograms of red flowers of *Rhododendron schlippenbachii* Maxim.

No. [1)]	Compound	RT [2)]	RRT [3)]	Selected ion for quantification [4)]
1	Lactic acid	4.808	0.442	147
2	Valine	5.215	0.480	146
3	Glycolic acid	6.408	0.590	147
4	Serine	6.968	0.641	116
5	Ethanolamine	7.050	0.649	174
6	Glycerol	7.067	0.650	147
7	Proline	7.386	0.679	142
8	Nicotinic acid	7.417	0.682	180
9	Glycine	7.447	0.685	174
10	Succinic acid	7.518	0.692	147
11	Glyceric acid	7.618	0.701	147
12	Fumaric acid	7.857	0.723	245
13	Threonine	8.140	0.749	219
14	*β*-Alanine	8.565	0.788	174
15	Malic acid	9.092	0.836	147
16	Aspartic acid	9.328	0.858	100
17	Pyroglutamic acid	9.430	0.868	156
18	4-Aminobutyric acid	9.452	0.870	174
19	Threonic acid	9.597	0.883	147
20	Glutamic acid	10.127	0.932	246
21	Phenylalanine	10.258	0.944	218
22	*p*-Hydroxybenzoic acid	10.272	0.945	223
23	Xylose	10.343	0.952	103
24	Asparagine	10.533	0.969	116
IS	Ribitol (Internal Standard)	10.867	1.000	217
25	Vanillic acid	11.258	1.038	297
26	Glutamine	11.303	1.040	156
27	Citric acid	11.580	1.065	273
28	Quinic acid	11.838	1.089	345
29	Fructose	11.948	1.099	103
30	Glucose	12.107	1.114	147
31	Mannose	12.260	1.128	147
32	Inositol	13.392	1.232	305
33	Ferulic acid	13.502	1.242	338
34	Tryptophan	14.237	1.310	202
35	Sinapic acid	14.393	1.324	338
36	Sucrose	16.350	1.504	217
37	Maltose	16.858	1.551	147
38	Trehalose	16.883	1.553	191
39	Raffinose	20.070	1.846	217

[1)] RNumbers represent the compound index for the chromatogram peaks shown in Fig S1. [2)] Retention time (min). [3)] Relative retention time (retention time of the analyte/retention time of the IS). [4)] Specific mass ion used for quantification.
